# Bridging the Gap between Gut Microbial Dysbiosis and Cardiovascular Diseases

**DOI:** 10.3390/nu9080859

**Published:** 2017-08-10

**Authors:** Kimberley Lau, Varun Srivatsav, Ayesha Rizwan, Andrew Nashed, Rui Liu, Rui Shen, Mahmood Akhtar

**Affiliations:** 1 Bachelor of Health Sciences (Honours), Faculty of Health Sciences, McMaster University, Hamilton, ON L8S 4L8, Canada; kimberley.lau@learnlink.mcmaster.ca (K.L.); varun.srivatsav@medportal.ca (V.S.); ayesha.rizwan02@gmail.com (A.R.); andrewnashed@hotmail.com (A.N.); rui.liu@learnlink.mcmaster.ca (R.L.); rui.shen@learnlink.mcmaster.ca (R.S.); 2 MD Program, Faculty of Medicine, University of Toronto, Toronto, ON M5S 1A8, Canada; 3 Michael G. DeGroote School of Medicine, Faculty of Health Sciences, McMaster University, Hamilton, ON L8S 4K1, Canada; 4 Sr. Principal Scientist, Research Executive Administration, King Fahad Specialist Hospital, Dammam 32253, Saudi Arabia

**Keywords:** gut microbiota, dysbiosis, cardiovascular disease, probiotics, prebiotics

## Abstract

The human gut is heavily colonized by a community of microbiota, primarily bacteria, that exists in a symbiotic relationship with the host and plays a critical role in maintaining host homeostasis. The consumption of a high-fat (HF) diet has been shown to induce gut dysbiosis and reduce intestinal integrity. Recent studies have revealed that dysbiosis contributes to the progression of cardiovascular diseases (CVDs) by promoting two major CVD risk factors—atherosclerosis and hypertension. Imbalances in host–microbial interaction impair homeostatic mechanisms that regulate health and can activate multiple pathways leading to CVD risk factor progression. Dysbiosis has been implicated in the development of atherosclerosis through metabolism-independent and metabolite-dependent pathways. This review will illustrate how these pathways contribute to the various stages of atherosclerotic plaque progression. In addition, dysbiosis can promote hypertension through vascular fibrosis and an alteration of vascular tone. As CVD is the number one cause of death globally, investigating the gut microbiota as a locus of intervention presents a novel and clinically relevant avenue for future research, with vast therapeutic potential.

## 1. Introduction

### 1.1. Definition and Introduction to the Gut Microbiota

The gut microbiota is a collection of microbial populations such as bacteria, fungi, viruses, and parasites, that reside within the gut, otherwise known as the gastrointestinal (GI) tract [1]. Over 100 trillion microbial cells and 1000 different bacterial species comprise the environment of the human gut [2].

The GI tract is heavily colonized by a collection of gut microbiota, predominantly bacteria, that can be both beneficial and harmful to the host [3,4].

Gram-positive Firmicutes and Gram-negative Bacteroidetes comprise the majority of the species in a healthy adult gut [5]. Although the balance of these species is variable between individuals, it remains relatively constant within a healthy individual [5].

### 1.2. Establishment, Development, and Changes to the Gut Microbiota

The microbial composition is host-specific, changing throughout an individual's lifetime in response to endogenous and exogenous factors. Establishment of the microbiota begins well before birth, which is evident from the assortment of microbiota found in the meconium [6].

The process of birthing introduces a variety of microbes [5]. Vaginal delivery may have the biggest impact on the diversity and development of the gut microbiota, serving as the primary inoculum [3,7].

The human vagina is comprised primarily of *Lactobacillus* and *Prevotella*, which are passed on through vertical inheritance to the child. However, with many babies being born through a cesarean section (C-section) mode of delivery, the initial bacterial profile of the baby is modified. Instead, the bacterial composition is quite similar to that found on human skin, composed of *Staphylococcus*, *Corynebacterium*, and *Propionibacterium*. Studies have even demonstrated that these bacteria are not closer in composition to the mother’s skin, but rather the skin of the health professionals which the baby may encounter during the delivery [5]. A C-section may therefore make an individual further susceptible to pathogens; one study found that 64–82% of methicillin-resistant *Staphylococcus aureus* infections in newborns occurs in C-section-delivered infants [8].

Starting from childbirth, the host is exposed to factors that influence gut microbiota composition and increase its diversity [3,7]. The variety of microbial species increases rapidly as the infant ages, which could be due to encountering different bacterial species with exposure to different foods and surfaces [9]. As the infant ages, ingestion of complex, plant-based foods causes an increase in bacteria associated with an adult gut, such as Bacteroidetes, as well as a more stable bacterial community [10].

Diet also plays a huge role in the establishment of certain populations of bacteria, especially during the early ages of life [5]. Starting at birth, breastfeeding may play a positive role in influencing protective bacterial populations, such as bifidobacteria [11]. Diet may also influence certain individuals to gain bacteria that are more suited in helping to digest the regular foods that areconsumed [5]. For instance, a study of rural children in Burkina Faso in Africa found a high abundance of bifidobacteria, useful in digesting the plant-rich diets of these children [12]. The bacterial colonization process is therefore extremely unstable and rapidly changing in an infant, only stabilizing and approaching an adult state after the first 1–3 years of life [5].

### 1.3. Homeostatic Functions

The gut microbiota is vital in the maintenance of host homeostatic functions through involvement in digestion, metabolism and the gut’s immune system. Most of the food consumed is digested by human enzymes before getting absorbed by the small intestine, however, gut microbes contribute to the digestion of complex dietary fibers [13]. With respect to metabolism, the gut microbiota plays a significant role in the breakdown of complex carbohydrates that cannot be hydrolyzed by host enzymes [4,14]. The complex carbohydrates are fermented into short chain fatty acids (SCFAs), primarily butyrate, propionate, and acetate, which serve many functions in the body. Butyrate can regulate gene expression in colonocytes, and is also important in energy and glucose homeostasis [4]. Acetate serves a function in fat regulation and storage, while propionate is taken up by the liver and is used as a substrate for gluconeogenesis [4,15]. Interestingly, one of the studies showed that all three SCFAs are interchangeable by gut microbes according to their requirement of a specific SCFA [16].

In addition to their integral role in metabolism, the gut microbiota also plays a significant role in immune function. For example, one of the main contributions of microbiota to the host is the development of the gut-associated lymphoid tissues (GALTs), which are a mucosa-associated tissue that line the gut and induce immune responses [1,17]. The gut microbiota stimulates the development of isolated lymphoid follicles, which are organized lymphoid structures in the small intestine, through pattern recognition receptor (PRR) activation [1,17]. PRRs are expressed on immune cells, such as macrophages and dendritic cells (DCs). These receptors recognize pathogen-associated molecular patterns (PAMPs) on microbes to initiate an immune response [1,3]. Like pathogens, the gut microbiota also has PAMPs that are recognized by different classes of PRR such as Toll-like receptors (TLRs) and nucleotide-binding-oligomerization-domain receptors. The gut microbiota is involved in the activation and differentiation of various T and B lymphocytes [1,3]. In addition, the gut microbiota modulates the mucosal production of Immunoglobulin A, which plays a critical role in maintaining intestinal barrier function [1,3].

### 1.4. Gut Dysbiosis

Gut dysbiosis is an alteration in the composition of the gut microbiota that can result from exposure to several factors such as diet, increased stress or levels of inflammatory markers, and antibiotic usage [18]. The alteration in microbial flora may explain why some individuals are more prone to develop certain diseases [19]. The relationship between the microbial composition and disease predisposition is not a cause-and-effect relationship, but the microbiome is the main contributor in many disease conditions, an avenue which is recently gaining the attention of the scientific community [20]. Gut dysbiosis can disturb various homeostatic functions of the human body and play a significant role in the pathophysiology of a variety of metabolic diseases. In fact, dysbiosis or changes in microbial composition are increasingly linked to several non-communicable diseases including diabetes [21], obesity [22,23], cancer [22,24], allergic asthma [25,26], and others.

Microbial compositions or dysbiosis patterns differ in different disease conditions. Recently Emoto et al. reported a characteristic change in microbial composition in coronary artery disease patients, where there was a significant increase in *Lactobacillales* (Firmicutes) and a decrease in Bacteroidetes [27]. While in case of type 2 diabetes, patients showed a decrease in Firmicutes as well as a non-significant increase in Bacteroidetes and Proteobacteria [28].

Dysbiosis can be implicated in the development of cardiovascular diseases (CVDs), a group of disorders affecting the heart and blood vessels [29,30]. CVDs represent approximately 31% of all global deaths, 80% of which occur in low- and middle-income countries [31]. The prevalence of CVDs worldwide demonstrates the pertinence of investigating their pathophysiology, in light of creating better therapeutic targets. Gut dysbiosis presents a promising avenue for such research, as it plays a major role in the progression of atherosclerosis and hypertension, two major risk factors for CVDs. This review will discuss the contribution of dysbiosis to atherosclerosis and hypertension, and how these risk factors can progress to CVDs, before proposing therapeutic strategies that target dysbiosis.

## 2. Atherosclerosis

Atherosclerosis is a chronic inflammatory disease characterized by the formation of plaque in arteries, which consists of necrotic cores, calcified regions, and an accumulation of lipids and cells such as leukocytes, foam cells, and endothelial cells (ECs) [29,30]. Gut dysbiosis can contribute to the development and progression of atherosclerosis through two major pathways—the metabolism-independent pathway and the metabolism-dependent pathway [32].

### 2.1. Metabolism-Independent Pathway

Gut dysbiosis can be involved in the pathogenesis of atherosclerosis directly through the metabolism-independent pathway [32]. Specifically, bacterial components such as lipopolysaccharides (LPS) found on the outer membrane of Gram-negative bacteria can promote the formation of foam cells, which are a major component of atherosclerotic plaque [33].

Foam cells are macrophages, phagocytic immune cells, that have engulfed excessive amounts of modified low density lipoprotein (LDL) cholesterol in an attempt to remove it from the bloodstream [34,35]. LDLs are responsible for the transport of cholesterol within the bloodstream [36]. The formation of foam cells is initiated when apolipoprotein B on the surface of circulating LDLs binds to LDL receptors on the endothelium, initiating endocytosis of the LDL into the tunica intima [34]. The LDL then undergoes oxidation through enzymatic attack or reaction with reactive oxygen species to produce oxidized, low density lipoproteins (oxLDLs) [37]. The accumulation of oxLDLs within the arterial wall stimulates ECs to express cell adhesion molecules such as vascular cell adhesion molecule-1 (VCAM-1) and chemokines such as monocyte chemoattractant protein-1 (MCP-1), that cause monocytes to adhere to the endothelium and migrate into the tunica intima respectively [34]. The oxLDLs also stimulate the production of macrophage-colony-stimulating factor (M-CSF) that induces differentiation of the incoming monocytes into mature macrophages [34]. Scavenger receptors (ScRs), such as cluster of differentiation 36 (CD36) that are expressed on the surface of macrophages, then mediate the uptake of oxLDL into the macrophage [34,38]. The accumulation of modified cholesterol within the macrophages leads to foam cell formation [34]. The foam cells deposit in arterial plaque, further contributing to atherosclerosis [34].

The body has internal homeostatic mechanisms such as reverse cholesterol transport (RCT), to counteract the accumulation of excess cholesterol in peripheral tissues. RCT is a process by which excess cholesterol is brought to the liver to be converted into bile acids (BAs) [39,40,41]. This transport is specifically mediated by the apolipoprotein A1 (ApoA-1) on high density lipoproteins (HDLs) which bind to cholesterol to facilitate transport to the liver [39,40,41]. Inside the macrophages, this process is mediated by a variety of receptors, specifically the liver X receptors (LXRs) α and β, and the cholesterol transporters adenosine triphosphate (ATP)-binding cassette transporter A1 (ABCA1) and ATP-binding cassette transporter G1 (ABCG1) [41,42]. When macrophages uptake oxLDLs, LXRs are stimulated and bind to LXR response elements on DNA to increase the expression of cholesterol transporters such as ABCA1 and ABCG1 [41,42]. The end result of this transcriptional cascade is oxLDL being removed from the cell, transported to the liver, and subsequently excreted through BAs [41,42]. This efflux of cholesterol from foam cells is a critical step in preventing the development of atherosclerotic plaque.

Gut dysbiosis can overwhelm mechanisms such as RCT and promote the formation of foam cells, specifically by inducing metabolic endotoxemia [41,43,44,45]. Metabolic endotoxemia is a condition characterized by an increased presence of LPS in circulation [33,46]. High-fat (HF) diet-induced dysbiosis is associated with reduced presence of bifidobacteria, which normally promote intestinal barrier function and prevent bacterial translocation [33]. Dysbiosis also results in the reduced expression of intestinal tight junction proteins, further increasing intestinal permeability [46]. This allows for increased levels of LPS to enter circulation, which goes on to promote inflammation and foam cell formation, by acting on TLR4 [33]. TLR4 is a PRR expressed on cells such as macrophages, ECs, enterocytes, and DCs [33]. Circulating LPS are sensed by a cell-surface-receptor complex that contains TLR4 and its co-receptors cluster of differentiation 14 (CD14) and myeloid differentiation protein-2(MD-2) [47]. In response to LPS-binding, the intracellular domain of TLR4 activates several signal transduction responses that lead to the production of pro-inflammatory cytokines, chemokines, and cell-adhesion molecules [33,47,48,49]. One of these complex transduction responses involves the molecule myeloid differentiation primary response gene 88 (MYD88) and will be explored in detail below.

#### MYD88 Signaling

**Figure 1:**
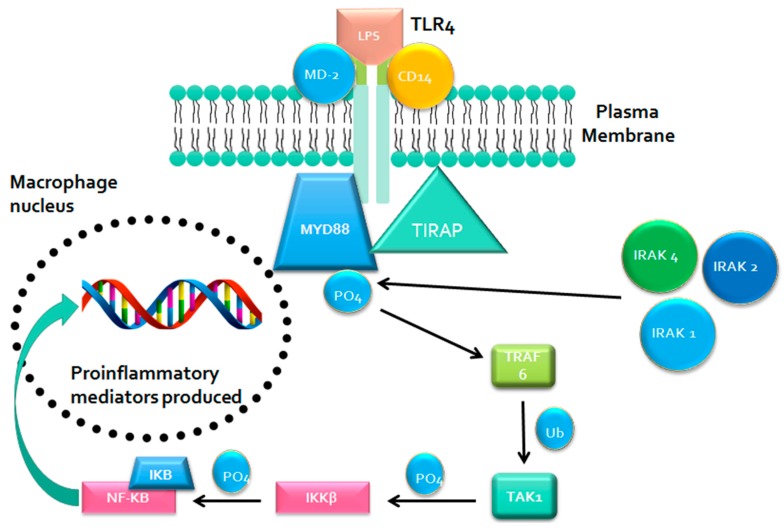
MYD88-mediated TLR4 signaling pathway results in the production of pro-inflammatory cytokines. Activation of TLR4 by LPS through MYD88 dependent pathway results in activation and nuclear translocation of NF-κB which upregulates production of pro-inflammatory cytokines and chemokines. Abbreviations: MYD88 - myeloid differentiation primary response gene 88, TLR4—Toll like receptor 4, LPS—lipopolysaccharides, TIRAP—toll/interleukin-1 receptor domain-containing adaptor protein, MD-2—myeloid differentiation protein-2, CD14—cluster of differentiation 14, IRAK—interleukin-1 receptor-associated kinase, TRAF—tumour necrosis factor receptor associated factor, TAK—transforming growth factor-beta-activated kinase, NF-κB – nuclear factor kappa B.

This signaling pathway involves the activation of the sorting adaptor molecule toll/interleukin-1 receptor (TIR)-domain-containing adaptor protein (TIRAP) that recruits MYD88 to the intracellular domain (Figure 1) [48]. The MYD88 then recruits the interleukin-1 (IL-1) receptor-associated kinase 1 (IRAK-1), IRAK-2, and IRAK-4, which will phosphorylate and activate the tumor necrosis factor (TNF) receptor-associated factor 6 (TRAF6) [47]. TRAF6 will add ubiquitin to the transforming growth-factor-beta-activated kinase 1 (TAK1), allowing TAK1 to bind to and phosphorylate the inhibitor of kappa B kinase-beta (IKKβ) [47]. The IKKβ will then phosphorylate the inhibitor of kappa B (IκB) [47]. Normally, IκB is bound to the transcription factor nuclear factor kappa B (NF-κB), sequestering it within the cytoplasm and preventing its nuclear translocation [33]. However, phosphorylation of IκB causes its degradation, thereby allowing NF-κB to enter the nucleus and increase the expression of pro-inflammatory cytokines such as IL-6, IL-1β, IL-27, and tumor necrosis factor-alpha (TNF-α), chemokines such as MCP-1 and cell adhesion molecules such as VCAM-1 [33,47,48,49].

**Figure 2:**
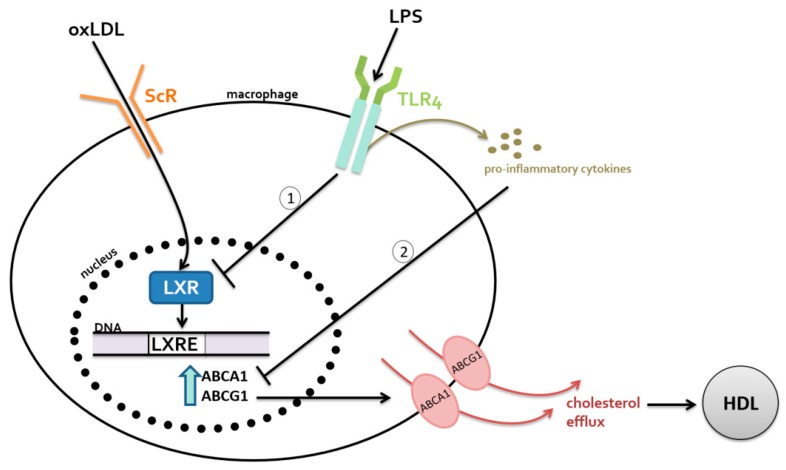
Dysbiosis can induce metabolic endotoxemia, an increased presence of lipopolysaccharides (LPS) in circulation. The LPS-mediated interference of reverse cholesterol transport (RCT) in macrophages occurs (1) directly by reduced expression of LXR and (2) indirectly through mechanisms that upregulate pro-inflammatory cytokines. Abbreviations: oxLDL—oxidized low density lipoprotein, HDL—high density lipoprotein, LXR—liver X receptor, LXRE – Liver X Receptor response element, ScR – scavenger receptor, TLR4 – Toll like receptor 4, ABCA1— adenosine triphosphate (ATP)-binding cassette (ABC) cholesterol transporters including ABC subfamily A member 1.

The increased LPS-induced expression of chemokines and cell adhesion molecules contributes to atherosclerosis progression by promoting monocyte adhesion to the endothelial layer and initiating the process of foam cell formation [33,43,44,45,49,50,51,52,53]. Additionally, LPS binding to TLR4 directly inhibits LXRs, which then reduces the expression of ABCA1 and ABCG1 [41,43,44,45]. LPS has also been shown to inhibit the expression of cholesterol transporters indirectly through the release of pro-inflammatory cytokines [41]. When macrophages were incubated with cytokines such as TNF-α and IL-1β, the mRNA levels of the cholesterol transporters ABCA1 and ABCG1 were significantly reduced [41]. Figure 2 summarizes the direct and indirect ways that gut microbiota dysbiosis contributes to the pathogenesis of atherosclerosis.

### 2.2. Metabolism-Dependent Pathway

In addition to the metabolism-independent pathway, dysbiosis can exert pro-atherosclerotic effects by altering the generation of a variety of metabolites. In particular, dysbiosis has been shown to affect the metabolism of bile acids (BAs), and the production of trimethylamine-n-oxide (TMAO), and butyrate [32].

#### 2.2.1. Bile Acids

BAs are synthesized from cholesterol, and constitute a major pathway for cholesterol catabolism [54]. In the context of atherosclerosis, BAs have two important functions. Their synthetic pathway acts as a major route for cholesterol elimination, and secondary BAs exhibit significant athero-protective effects [55]. Gut microbiota regulate BA metabolism through their bacterial bile-salt hydrolase (BSH) activity, which is essential for the formation of secondary BAs [55]. A dysbiotic microbial composition can possess decreased BSH activity, exerting a variety of pro-atherosclerotic effects. Specifically, dysbiosis can lead to impaired cholesterol elimination, and the net effect of this is the progression of atherosclerotic plaque. Primary BAs are synthesized from cholesterol in the liver, and are excreted into the small intestine to aid lipid emulsification [54]. Within the intestinal lumen, gut microbiota can catalyze the deconjugation of primary BAs to form secondary BAs through bacterial BSH activity [4]. This results in a pool of primary and secondary BAs, predominantly comprising primary BAs. BA transporters within the terminal ileum reabsorb 95% of BAs, which are then recycled in the liver to be secreted again [54]. Since secondary BAs are less soluble, they are less likely to be reabsorbed. Thus, they are more likely to be excreted, providing a pathway for cholesterol elimination [56]. This intricate cycle constitutes the enterohepatic circulation of BAs, which is tightly governed by BA-signaling of the hepatic farnesoid X receptor (FXR) [54].

**Figure 3:**
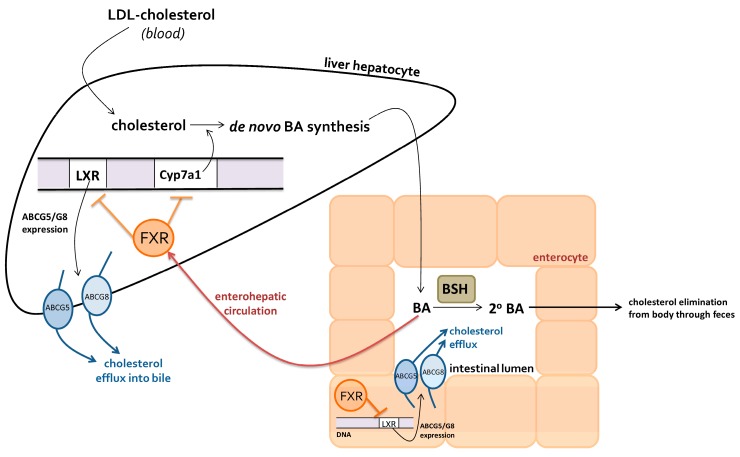
Regulation of cholesterol elimination through bacterial bile-salt hydrolase (BSH) mediated bile acids (BA) activation of hepatic farnesoid X receptor (FXR).

The activation of FXR by BAs, and subsequent signaling pathways, is directly influenced by BSH activity. BSH promotes cholesterol elimination through increased levels of secondary BAs, which are more likely to be excreted in feces than primary BAs [56]. Additionally, BSH prevents systemic cholesterol accumulation by promoting the de novo synthesis of BAs and increased cholesterol efflux by enterocytes and hepatocytes [55]. The process of removal of cholesterol through BSH is depicted in Figure 3.

Decreased BSH activity due to dysbiosis leads to less primary BA conversion and therefore more BAs being re-absorbed into the enterohepatic circulation. This causes an increased activation of hepatic FXR, which inhibits the expression of the BA synthetic enzyme cholesterol 7 alpha-hydroxylase (Cyp7a1), and the nuclear receptor LXR [55]. Greater inhibition of Cyp7a1 results in decreased synthesis of BAs, and consequently, lower serum cholesterol uptake by the liver. LXR expression normally promotes the upregulation of cholesterol transporters ABCG5/G8, increasing cholesterol efflux from hepatocytes and enterocytes [55]. Therefore, FXR-driven LXR inhibition results in the downregulation of ABCG5/G8, promoting cholesterol accumulation within the liver and intestinal cells. Due to its role in regulating BA signaling, reduced bacterial BSH activity can lead to the accumulation of cholesterol, promoting the formation of foam cells, and ultimately, atherosclerotic plaque [34].

#### 2.2.2. Trimethylamine-N-Oxide

In addition to being involved in the metabolism of BAs, the gut microbiota plays an integral role in the production of TMAO—a metabolite derived primarily from dietary phosphatidylcholine and L-carnitine [32]. Both TMAO precursors are found commonly in HF food products such as red meat, cheese, and eggs [57]. Higher baseline levels of TMAO have been directly linked to the increased occurrence of major adverse cardiovascular events, demonstrating the significance of TMAO as a predictor of cardiovascular risk [58].

The gut microbiota has been shown to participate in the formation of TMAO through the production of the precursor trimethylamine (TMA) [59]. Recent studies have identified two distinct classes of bacterial enzymes, choline-specific and carnitine-specific TMA lyases, that cleave a carbon-nitrogen bond to form TMA [59]. Once absorbed into the bloodstream, TMA is transported to the liver where it is subsequently converted into TMAO by hepatic flavin-containing monooxygenases (FMOs) [59].

Different bacterial compositions have varying abilities to generate TMAO. Thus, it can be hypothesized that a dysbiotic microbial composition containing more TMA-producing bacteria can lead to higher levels of TMAO and increased CVD risk [58]. For instance, TMAO levels have been found to correlate with certain human gut microbial enterotypes. Higher TMAO plasma concentrations were found to be associated with the *Prevotella* enterotype, as opposed to the *Bacteroides* enterotype [59].

TMAO promotes the development of atherosclerosis by impairing RCT and cholesterol catabolism. TMAO has been found to increase the expression of the macrophage ScR CD36 [59]. This upregulation promotes foam cell formation by ScR-driven oxLDL uptake [34]. TMAO has also been shown to decrease the expression of the hepatic BA synthetic enzymes Cyp7a1 and Cyp27a1 [59]. Down-regulating BA synthetic enzymes results in decreased elimination of cholesterol and decreased RCT [54]. Overall, the most significant effect of increased TMAO levels is systemic cholesterol accumulation, leading to an increased generation of atherosclerotic plaque.

#### 2.2.3. Butyrate

Butyrate, one of the primary SCFAs produced by bacterial fermentation of non-digestible carbohydrates, has a variety of athero-protective effects [32]. In addition to its homeostatic functions, butyrate has also been shown in studies to induce anti-inflammatory effects and to reduce monocyte adhesion to the endothelium [60,61]. Butyrate has been shown to inhibit the translocation of NF-kB, and thereby reduce the expression of pro-inflammatory cytokines and TNF-α-induced VCAM-1 expression [60,61]. Reduced expression of VCAM-1 leads to decreased monocyte adhesion to the endothelium, hindering foam cell formation [60].

Gut dysbiosis in atherosclerosis revealed a reduced population of butyrate producers such as Eubacterium and Roseburia [62,63]. Decreased butyrate levels can result in an attenuation of its anti-inflammatory effects and the increased adhesion of monocytes to the inflamed endothelium, promoting plaque development [60,61].

## 3. Hypertension

In addition to atherosclerosis, dysbiosis can contribute to the progression of hypertension, another key risk factor for CVD. Hypertension can be defined as small decreases in the arterial lumen that increase peripheral vascular resistance, resulting in high blood pressure (BP) [64]. In particular, gut dysbiosis contributes to hypertension through oxLDL-induced vasoconstriction [65].

### OxLDL and Vasoconstriction

The mechanisms of vasoconstriction and vasodilation are crucial to the regulation of BP [65]. Studies have demonstrated that patients afflicted with chronic heart failure and coronary artery diseases have decreased vasodilators and increased vasoconstrictors, indicating the importance of these compounds in mediating the pathophysiology of CVDs [66,67]. As previously described in our discussion on atherosclerosis, dysbiosis can promote pro-inflammatory cytokine expression and foam cell formation. Inflammation can induce oxidative stress and vice versa, creating a positive feedback loop that fosters an increasingly oxidative environment [68]. This elevated oxidative stress can stimulate oxidation of LDL [68]. Higher levels of oxLDL can cause an underproduction of vasodilators and an overproduction of vasoconstrictors, leading to hypertension [65,66].

**Figure 4:**
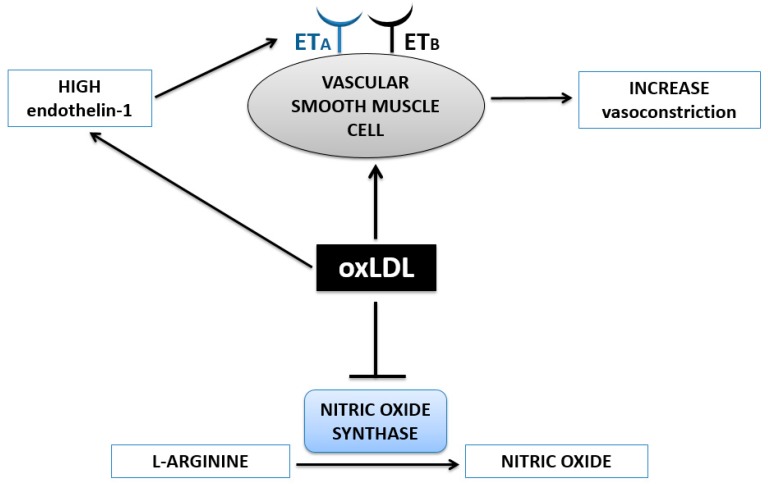
Accumulation of oxidized, low density lipoprotein (oxLDL) in plaque induces vasoconstriction in two ways – upregulation of endothelin-1 expression and inhibition of nitric oxide synthase enzyme. Abbreviations: ET_A_—endothelin receptor A, ET_B_—endothelin receptor B.

OxLDL tips the balance between vasoconstrictors and vasodilators through two mechanisms (Figure 4) [66,69,70]. One of the mechanisms involves nitric oxide (NO), an important vasodilator in the body [65,69]. NO synthase oxidizes L-arginine to form NO [65]. However, oxLDL is associated with the inhibition and decreased activity of NO synthase [65,69]. The subsequent decreased production and release of NO leads to less vasodilation, contributing to hypertension [65,69].

In addition, oxLDL can stimulate an increased production of endothelin-1 to exert a vasoconstrictive effect [65]. At low concentrations, endothelin-1 is a vasodilator and activates endothelin receptor B (ET_B_) on ECs [65]. This promotes NO release and subsequent vasodilation [65]. However, accumulation of oxLDL within plaque can activate a vasoconstrictive pathway by inducing a higher expression of endothelin-1 [65]. Specifically, oxLDL stimulates VSMCs to also produce and release endothelin-1 [65,70]. The higher levels of endothelin-1 then act primarily on endothelin receptor A (ET_A_) on VSMCs to induce vasoconstriction, resulting in a hypertensive effect [65].

## 4. Treatments

### 4.1. Dysbiosis Treatment

In pursuit of effective CVD treatments, the gut microbiota is a promising avenue for research. Rather than treating the symptoms associated with CVDs, the newly elucidated connections between dysbiosis and CVD pathogenesis present novel opportunities for early intervention. Several treatment routes have been proposed to correct the dysbiotic gut microbial compositions that promote CVDs. These options include the administration of pre- and probiotics to fertilize and restore the gut with beneficial microbial populations (Table 1).

### 4.2. Prebiotics

Among different environmental factors affecting the human microbiota, diet is a major factor which shapes the gut microbiota [71]. Prebiotics are dietary constituents that can cause specific changes in the composition and activity of intestinal microbiota to elicit beneficial effects on the host [72]. Prebiotics can incur atheroprotective effects and reduce the risk of CVDs by selectively promoting the growth of beneficial gut microbiota [73].

Plant polyphenols found in fruits and vegetables have shown to have prebiotic properties [74,75]. These bioactive polyphenols travel through the gut without being modified, and have been able to influence gut microbiota composition [74]. The prebiotic effects of apples have been of particular interest in the research community due to their frequent consumption in society. The modification of microbiota composition through apple components along with the direct effect of polyphenols may have cardioprotective effects, such as reducing inflammation [76] and total cholesterol levels [74,77].

Inulin (IN) and oligofructose (OF) are polydisperse fructans that have prebiotic properties well-established in the literature [78]. Since host pancreatic enzymes cannot hydrolyze their β-2,1 glycosidic linkages, these exogenous polysaccharides are able to pass through the small intestine completely undigested, making them readily available for gut microbiota metabolism [78]. Specifically, IN and OF are used as energy substrates by Bifidobacterium spp. that express β-fructofuranosidase [78]. These enzymes catalyze the depolymerization of fructans to derive monosaccharides, which go on to stimulate bifidobacteria growth [79].

Bifidobacteria growth can help maintain the integrity of the intestinal barrier. Unlike pathogenic bacteria, bifidobacteria do not degrade intestinal mucus glycoproteins [80]. In addition, they promote the endogenous production of glucagon-like-peptide 2 (GLP-2) [80]. GLP-2 regulates the expression of tight junction proteins to reduce intestinal permeability and prevent the translocation of harmful elements into circulation [81]. Significantly reduced bifidobacteria levels in dysbiosis can lead to an increase in bacterial translocation and metabolic endotoxemia through increased intestinal permeability [80]. The size of the bifidobacteria population has been found to be inversely correlated with serum levels of LPS [80]. Therefore, prebiotic treatments, such as dietary fructan supplements, that enhance bifidobacteria populations can reduce LPS translocation into circulation, preventing the atherosclerotic effects of metabolic endotoxemia.

Furthermore, fermentation of dietary fructans by bifidobacteria results in increased SCFA production [78]. Long-term studies have shown that OF-enriched prebiotic supplementation in rats increased cecal SCFA levels, particularly those of butyrate [78]. As mentioned previously, dysbiosis in atherosclerosis can lead to a reduced presence of the butyrate producers *Eubacterium* and *Roseburia* [62,63]. Prebiotic administration of IN-type fructans can promote the restoration of butyrate producing populations and, in turn, the atheroprotective effects of butyrate [78].

### 4.3. Probiotics

Probiotics can be defined as “live microorganisms that, when administered in adequate amounts, confer a health benefit on the host” [82]. The most common probiotics are lactobacilli and bifidobacteria strains—natural residents of the human gut [83]. It is important to emphasize that the health effects of probiotics are strain specific. Current research on probiotics has heavily focused on lactobacilli strains [84]. This is likely due to the high BSH activity found in the species. In addition to possessing a plethora of atheroprotective effects, BSH also detoxifies bile salts to enhance intestinal survivability [85]. This confers lactobacilli strains an advantage in colonizing the human gut.

Probiotics present a novel therapy for treating elevated serum cholesterol levels [86]. By lowering circulating cholesterol, probiotics can impair the formation, progression, and eventual rupture of atherosclerotic plaque [86]. There are few double blinded, placebo-controlled clinical trials that can conclusively state the efficacy of probiotics in modulating human metabolism [86]. Many human clinical studies have yielded mixed results, with some studies finding no lipid-lowering effects [87]. However, a review of meta-analyses of high-quality randomized control trials has highlighted promising strains that elicit lipid-lowering effects [86].

Jones et al. conducted two double-blinded randomized control trials to assess the hypocholesterolemic effects of *Lactobacillus reuteri* strain NCIMB 30242 [88]. They found that *L. reuteri* elicited reductions in systemic cholesterol levels through their BSH activity [88]. Microencapsulation administration in yogurt formulation of *L. reuteri* decreased LDL-cholesterol (LDL-C) by 8.92%, serum total cholesterol (TC) by 4.81% and non-HDL cholesterol by 6.01% [88]. Furthermore, a second study by the same investigators demonstrated that the administration of probiotics in a lyophilized form was able to reduce LDL-C by 11.64%, TC by 9.14%, and non-HDL cholesterol by 11.3% [88]. Fuentes et al. also demonstrated the lipid-lowering effects of *Lactobacillus plantarum* CECT 7527, 7528, and 7529 in a double-blinded randomized control trial that showed a reduction in TC by 13.6% [84]. Overall, *L. reuteri* NCIMB 30242 and *L. plantarum* CECT 7527, 7528, and 7529 are amongst the most efficacious lipid-lowering strains, demonstrating their viability as a treatment option [89].

## 5. Conclusions

It is evident that gut microbiota isintegral to the homeostatic functions of the body, often having a protective role against disease [3]. Changes to the gut microbiota composition can lead to metabolic diseases such as CVDs, attenuating the protective homeostatic role that these microorganisms play [27,33].

**Figure 5:**
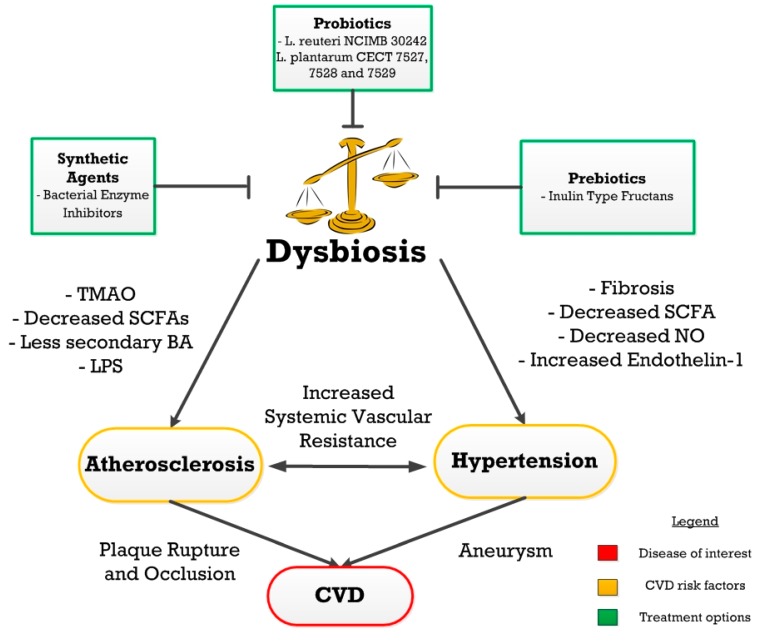
An overview of the relationships between dysbiosis, cardiovascular disease (CVD) risk factors, CVD, and potential treatments. Probiotics, prebiotics and synthetic agents can be used as a treatment for gut dysbiosis which can further prevent the progression of CVDs.

Dysbiosis plays a significant role in the development of CVDs, contributing to risk factors such as atherosclerosis and hypertension through inflammation, dyslipidemia, and arterial fibrosis (Figure 5) [32,34,50] Atherosclerosis is known to develop through two dysbiotic pathways, metabolism-independent and metabolite-dependent [32]. Metabolic endotoxemia leads to a pro-atherogenic inflammatory state in the metabolism-independent pathway [32,33,41,43,44,45,46]. On the other hand, the metabolite-dependent mechanism can contribute to atherogenesis through dysbiosis-induced alterations in levels of TMAO, secondary BAs, and butyrate [54,55,57,60,61]. Similarly, dysbiosis-induced alterations in vascular tone and fibrosis can lead to hypertension [50,65,66,67,69,70].

Although the associations between gut dysbiosis and CVDs are evident, research is currently underway to determine conclusively whether the linking pathways are causal, correlational, or consequential. Future scientific research should focus on fully elucidating the cellular mechanisms behind the dysbiotic pathways that contribute to CVDs. Despite the detrimental effects of dysbiosis, investigating potential treatment options has demonstrated that microbiota can also serve as the agent in re-establishing homeostasis. Further research should explore how prebiotics can aid the survival of probiotics to create effective synbiotic treatments for dysbiosis-induced CVDs. Although therapeutics for metabolic diseases have often targeted the host as an isolated entity, the scientific community should place a greater focus on exploring the therapeutic potential of the microorganisms that reside in the GI tract.

## Figures and Tables

**Table 1:** Summary of the effects of potential cardiovascular disease (CVD) treatments targeting gut microbiota composition, based on recent studies.

Treatment:	Prebiotics	Probiotics
Definition:	Dietary constituents that fertilize and promote healthy gut microbiota composition	Beneficial live microorganisms that can colonize the human gut to develop or restore healthy gut microbiota composition
Examples and Effects:	Plant polyphenols Fruits and vegetables (e.g., apples): reduce inflammation [76] and total cholesterol levels [74,77], promote bifidobacteria growth [75] Dietary fructans Foods high in inulin and/or oligofructose: promote bifidobacteria growth [90], restore butyrate-producing bacterial populations [91,92]	*Lactobaccillus* strains *L. reuteri* (microencapsulated in yogurt): reduce LDL-cholesterol, serum total cholesterol, and non-HDL cholesterol [88] *L. plantarum* (capsules): reduce serum total cholesterol [84]
